# Healthcare Pattern of Use Before and After Initiating a Long-Acting Antipsychotic Among a Cohort of 6221 Patients With a History of Psychosis

**DOI:** 10.1177/07067437261462697

**Published:** 2026-06-29

**Authors:** Emmanuel Stip, Josiane Courteau, Sébastien Brodeur, Yohann M. Chiu, Marie-Josée Fleury, Alain Lesage, Marc-André Roy

**Affiliations:** 1 Département de Psychiatrie et d'Addictologie, 12368 Universite de Montreal Faculte de medecine, Montreal, Canada; 2 Department of Psychiatry and Behavioral Science, College of Medicine and Health Science, United Arab Emirates University, Al Ain, United Arab Emirates; 3 Faculté de médecine et des sciences de la santé, Centre de recherche du Centre hospitalier universitaire de Sherbrooke (CRCHUS), Sherbrooke, Canada; 4 Département de Psychiatrie et Neurosciences, 12369 Université Laval, Québec, Canada; 5 Centre de Recherche CERVO, Université Laval, Québec, Canada; 6 Département de médecine de famille et de médecine d’urgence, Université de Sherbrooke, Sherbrooke, Canada; 7 Institut universitaire en santé mentale, Université McGill, Montréal, Canada; 8 Département de Psychiatrie, Université McGill, Montréal, Canada; 9 Centre de Recherche, Institut universitaire en santé mentale de Montréal (IUSMM), Montréal, Canada

**Keywords:** long-acting injectable antipsychotics, schizophrenia, health services utilization, community mental health, real-world cohort, registry, QAAPAPLE.

## Abstract

**Objective:**

To evaluate changes in antipsychotic treatment patterns and healthcare utilization before and after initiation of long-acting injectable antipsychotics (LAI-APs) in a large Québec population cohort, comparing individuals with schizophrenia (SCZ) to those with other psychotic disorders (non-SCZ).

**Method:**

We conducted a retrospective cohort study using linked Québec administrative databases (RAMQ, MED-ECHO, and public drug insurance) to identify 6,221 adults who initiated a LAI-AP between April 2013 and December 2016, after a 12-month LAI-free period. Participants were followed for 12 months before and after the index date. The cohort was stratified into SCZ and non-SCZ, and were further divided by regimen at initiation (LAI only; LAI + clozapine; LAI + other oral antipsychotic). Antipsychotic exposure and health-service usage (hospitalizations, emergency visits, outpatient and community care) trajectories were analyzed weekly using state-sequence analysis; pre- versus post-initiation comparisons used paired statistical tests.

**Results:**

Of 6,221 patients (63.4% male; mean age 41.6 years), initial treatments consisted of paliperidone LAI (55.7%), aripiprazole LAI (21.5%), risperidone LAI (6.9%), first-generation LAI (15.6%), and LAI combinations (0.2%); 40% received LAI only, 5% LAI + clozapine, 55% LAI + an oral antipsychotic. SCZ patients were more often male, economically disadvantaged, and more likely to receive clozapine. After LAI initiation, hospital days fell sharply by almost 70% and outpatient and community-care visits increased substantially. Use of oral antipsychotics decreased overall post-initiation, except for clozapine (which rose) and first-generation oral drugs (which remained stable).

**Conclusions:**

In this real-world Québec cohort, LAI-AP initiation was followed by a marked reduction in hospitalizations and a shift toward outpatient and community care, regardless of diagnosis. Observed differences in sociodemographic and clinical profiles between SCZ and non-SCZ patients—and among SCZ treatment subgroups—suggest the need for tailored care pathways. These findings support LAI-AP effectiveness in reducing healthcare utilization and inform resource planning.

## Plain Language Summary

How Injectable Medications Reduce Hospital Visits for Patients With Psychosis

A Quebec study of 6,221 patients reveals that starting long-acting injectable antipsychotics (LAIs) reduces hospital stays by 70% while increasing community-based follow-ups. These results demonstrate the effectiveness of LAIs in improving patient stability and highlight the need to tailor care according to the diagnosis, whether for schizophrenia or other psychotic disorders. This research supports the use of LAIs to optimize the use of healthcare services.

## Résumé

**Objectif:**

Évaluer l’évolution des schémas de traitement antipsychotiques et de l’utilisation des soins de santé avant et après l’instauration d’antipsychotiques injectables à action prolongée (APIAP) dans une vaste cohorte de la population au Québec, en comparant les personnes atteintes de schizophrénie (SCZ) à celles atteintes d’autres troubles psychotiques (non-SCZ).

**Méthodologie:**

Nous avons réalisé une étude de cohorte rétrospective à l’aide des bases de données administratives couplées du Québec (RAMQ, MED-ÉCHO et régime public d’assurance médicaments) afin d’identifier 6 221 adultes ayant commencé un traitement par APIAP entre les mois d’avril 2013 et de décembre 2016, après une période de 12 mois sans IAP. Les participants ont fait l’objet d’un suivi pendant 12 mois avant et après la date de référence. La cohorte a été stratifiée en deux groupes : SCZ et non-SCZ, puis subdivisée en fonction du schéma thérapeutique au moment de l’instauration du traitement (IAP seulement; IAP + clozapine; IAP + autre antipsychotique oral). Les trajectoires d’exposition aux antipsychotiques et d’utilisation des services de santé (hospitalisations, visites au service des urgences, soins ambulatoires et en milieu communautaire) ont été analysées sur une base hebdomadaire à l’aide d’une analyse de séquence d’états; les comparaisons avant et après l’instauration du traitement ont été effectuées à l’aide de tests statistiques appariés.

**Résultats:**

Parmi les 6 221 patients (63,4 % d’hommes; âge moyen de 41,6 ans), le traitement initial était la palipéridone IAP (55,7 %), l’aripiprazole IAP (21,5 %), la rispéridone IAP (6,9 %), IAP de première génération (15,6 %) et associations d’IAP (0,2 %); 40 % ont reçu un IAP seulement, 5 % ont reçu l’association IAP + clozapine, 55 % ont reçu l’association IAP + antipsychotique oral. Les patients du groupe SCZ étaient plus souvent des hommes, défavorisés sur le plan économique et plus susceptibles de recevoir de la clozapine. Après l’instauration de l’IAP, le nombre de jours d’hospitalisation a chuté de près de 70 %, et les visites en consultation externe et en soins communautaires ont augmenté considérablement. L’utilisation d’antipsychotiques oraux a diminué dans l’ensemble après l’instauration du traitement, à l’exception de la clozapine (qui a augmenté) et des antipsychotiques oraux de première génération (qui sont demeurés stables).

**Conclusions:**

Dans cette cohorte du monde réel au Québec, l’instauration d’un traitement par APIAP a été suivie d’une réduction marquée des hospitalisations et d’un virage vers les soins en consultation externe et en milieu communautaire, quel que soit le diagnostic. Les différences observées dans les profils sociodémographiques et cliniques entre les patients des groupes SCZ et non-SCZ, et entre les sous-groupes de traitement de la SCZ, indiquent la nécessité de mettre en place des parcours de soins personnalisés. Ces résultats confirment l’efficacité des APIAP pour réduire l’utilisation des soins de santé et orienter la planification des ressources.

## Introduction

Long-acting injectable antipsychotics (LAI-APs) play an increasingly vital role in the management of severe mental disorders by addressing treatment non-adherence, a critical risk factor for relapse, hospitalization, and elevated healthcare utilization.^1–4^ Although clinical guidelines typically view LAI-APs and oral antipsychotics as pharmacologically equivalent, this perspective is increasingly challenged by real-world studies consistently demonstrating greater effectiveness of LAI-APs in preventing relapse and reducing healthcare utilization.^5–8^

In prior work, Stip et al. have highlighted the real-world effectiveness of LAI-APs in Quebec's population using extensive provincial administrative databases, underscoring improvements in adherence, cost savings, and reduced healthcare utilization after LAI-AP initiation between 2008 and 2012.^9–13^ Their investigations within a national cohort of individuals diagnosed with schizophrenia (SCZ) spectrum disorders further confirmed these benefits, illuminating LAI-AP impact across diverse diagnostic categories.^14^

Building on this research, the present study introduces a large-scale retrospective population-based cohort of 6,221 individuals who initiated treatment with an LAI between 2013 and 2016, drawn from administrative sources within the Quebec universal healthcare system (RAMQ, MED ECHO, and drug insurance records). The database provided access to a population up to 2018; as this is a mirror study comparing 2 periods with an index date, the last patients included were in 2016. We evaluate healthcare utilization patterns both before and after initiation of a long-acting antipsychotic, encompassing variations across SCZ and non-SCZ groups.

The principal aims of this study are:

1. To characterize antipsychotic treatment trajectories surrounding LAI-AP initiation.
2. To quantify changes in healthcare service utilization—including inpatient stays, emergency department visits, and outpatient consultations—within a 1-year follow-up.
3. To compare these patterns between patients with a final diagnosis of SCZ versus those with other psychosis-related conditions

As a secondary objective, we stratified the SCZ and non-SCZ groups according to treatment at the index date, distinguishing patients receiving an LAI alone, an LAI in combination with clozapine, and an LAI combined with another oral antipsychotic.

By leveraging comprehensive administrative data, this analysis extends previous findings with more recent data by offering nuanced insights into trajectories of care across diagnostic subgroups, thereby informing tailored strategies for LAI-AP deployment and resource planning.

## Methods

### Design and Data Sources

Patients’ data for this population-based retrospective cohort study were acquired in Canada from the provincial health insurance board (*Régie de l’assurance maladie du Québec:* RAMQ), which manages universal health insurance for Québec residents, including coverage for most physicians and hospital services. This universal health program is complemented by a public prescription drug insurance plan (PPDIP) covering people aged 65 and over, all recipients of social welfare, and individuals who do not have access to a private drug insurance plan. Overall, around 45% of the general population of Québec is covered by the PPDIP, but results on the specific population with SCZ indicate that this sub-population was largely registered to the PPDIP.^15^ RAMQ owns and manages health administrative databases, including a hospital discharge register (MED-ECHO), patients’ demographic information, physicians’ reimbursement claims, and the provincial drug insurance plan. MED-ECHO contains information on dates of hospitalizations, non-pharmacological interventions, and principal and secondary diagnoses (according to ICD-9 before April 2006 and to ICD-10 thereafter). The RAMQ demographic databases provide information on patients’ age, sex, date of death, and eligibility for the PPDIP. The physician reimbursement claims database provides the date and diagnosis (according to ICD-9) associated with the service provided. The drug database contains information on the drugs claimed from community pharmacies by individuals covered by the PPDIP, including the date of delivery and the duration of the prescription. This drug database does not include information on inpatient drug treatment. Patient files were linked to provide demographic characteristics, medical and drug information using a unique encrypted identifier.

These data sources were used to create a database on severe mental disorders (DB SMD) including all people identified as suffering from a severe mental disorder (schizophrenia or schizoaffective disorders (SCZ), bipolar disorders, other psychoses) between 2002 and the end of 2017. This study was approved by the Research Ethics Board Committee of the Université de Sherbrooke.

### Study Cohort

From this large DB SMD, we extracted all patients starting at least one LAI-AP drug (with a clearance baseline period of 12 months without any LAI-AP) between April 1, 2013, and December 31, 2016 (Supplemental Figure S1). The index date refers to the date of LAI-AP initiation. Each new LAI-AP user was followed for 1 year and had to be continuously covered by the PPDIP during this follow-up period and during the baseline period of 1 year before index date.

The study cohort was divided into 2 groups: SCZ LAI-AP users and non-SCZ LAI-AP users. A patient was categorized as being in the SCZ group if their final recorded psychosis diagnosis was SCZ (ICD-9: 295; ICD-10: F20, F21, F23.3, F25). Otherwise, the patient was assigned to the non-SCZ group. These groups were further divided into 3 subgroups according to the initial treatment at index date: LAI only, LAI plus clozapine, and LAI plus another oral AP.

### Demographic and Clinical Characteristics

The following variables were assessed: sex (female/male); age at index date (continuous and categoric); low socioeconomic status (defined as senior with pension income supplement or being a recipient of social welfare); prescriber of the initial LAI-AP (psychiatrist/general practitioner/other physician); history of: depressive disorder (ICD-9: 311, 300.4; ICD-10: F32, F33), anxiety disorder (ICD-9: 300, except 300.4; ICD-10: F40-F49), substance-use disorder (ICD-9: 291, 292, 303-305; ICD-10: F10-F19), and personality disorder (ICD-9: 301; ICD-10: F60-F62); concomitant use of: lithium, antidepressants, divalproex, lamotrigine, benzodiazepine; and a physical health comorbidity index based on the validated combined Charlson-Elixhauser comorbidity index adapted for administrative databases.^16^

### Antipsychotics Utilization Trajectories

The following second-generation AP drugs were available during the study period: olanzapine (oral), risperidone (LAI and oral), quetiapine (oral; at least 300 mg per day), clozapine (oral), lurasidone (oral), paliperidone (LAI and oral), ziprasidone (oral), and aripiprazole (LAI and oral). First-generation AP (FGA) drugs were combined into 2 categories: FGA-LAI and oral FGA. Using the date of delivery and the duration of the prescription, a patient was considered exposed to the drug from the date(s) a prescription was claimed at a community pharmacy and for the duration the drug was provided. Hence, for each day of the follow-up period (1 year before and 1 year after), a patient was exposed to one or more of these AP or was not exposed to any AP, except for inpatient stays, as no information on drug treatment was available.

To represent the trajectory of AP use before and after the LAI-AP initiation, we included the following 9 states, in priority order: paliperidone LAI, aripiprazole LAI, risperidone LAI, FGA-LAI, more than 1 LAI-AP, oral SGA, oral FGA, both oral FAG and oral SGA, and no AP. This sequence of patient's exposure to consecutive AP is referred to the antipsychotic utilization trajectory.

### Healthcare Utilization Trajectories

Alongside the antipsychotic utilization trajectory, we considered the healthcare use trajectory before and after the LAI-AP initiation. These healthcare services included (referred to states), in priority order: (1) hospital stays, (2) emergency department visits, (3) outpatient visits, (4) ambulatory primary care visit, and (5) visits to local community service centres (French acronym CLSC). As before, the healthcare medical visit dates and the admission and discharge dates for the hospitalizations were used to produce a sequence of healthcare utilization before and after the LAI-AP initiation. In this case, we considered “week” as the time unit. If a patient has more than one medical visit during a given time unit (e.g., hospitalization and consultation in CLSC within the same week), the state with the highest priority, as listed above, was selected. This sequence of patient's healthcare utilization is referred to the healthcare trajectory.

### Statistical Analysis

We first described and compared the demographic, clinical characteristics, initial LAI-AP, and the specialty of the first LAI-AP prescriber among SCZ vs non-SCZ LAI-AP users and among the type of AP initiation for each group (LAI only vs. LAI + clozapine vs. LAI + another oral AP) (Chi-2 test for categorical variables and Kruskal–Wallis test for continuous variables). Then, the type of medication and the number of healthcare uses before and after LAI-AP initiation among the same groups were compared before and after index date (McNemar test for paired categorical variables and paired *t*-test for continuous variables).

**Figure 1.**
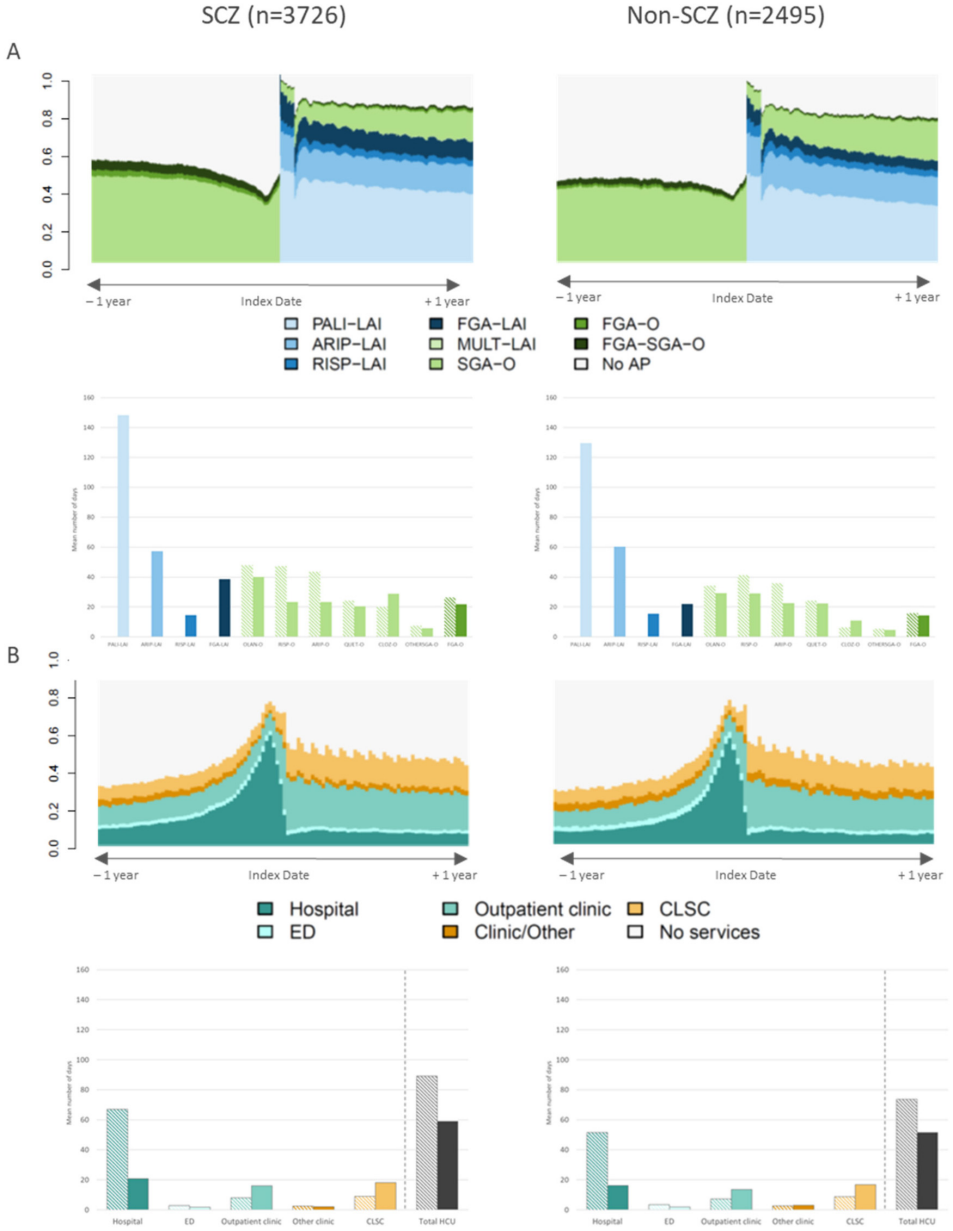
Antipsychotic trajectory (A) and healthcare use trajectory (B) before and after LAI-AP initiation among SCZ and non-SCZ: state distribution plots* and bar charts.^†^

**Figure 2.**
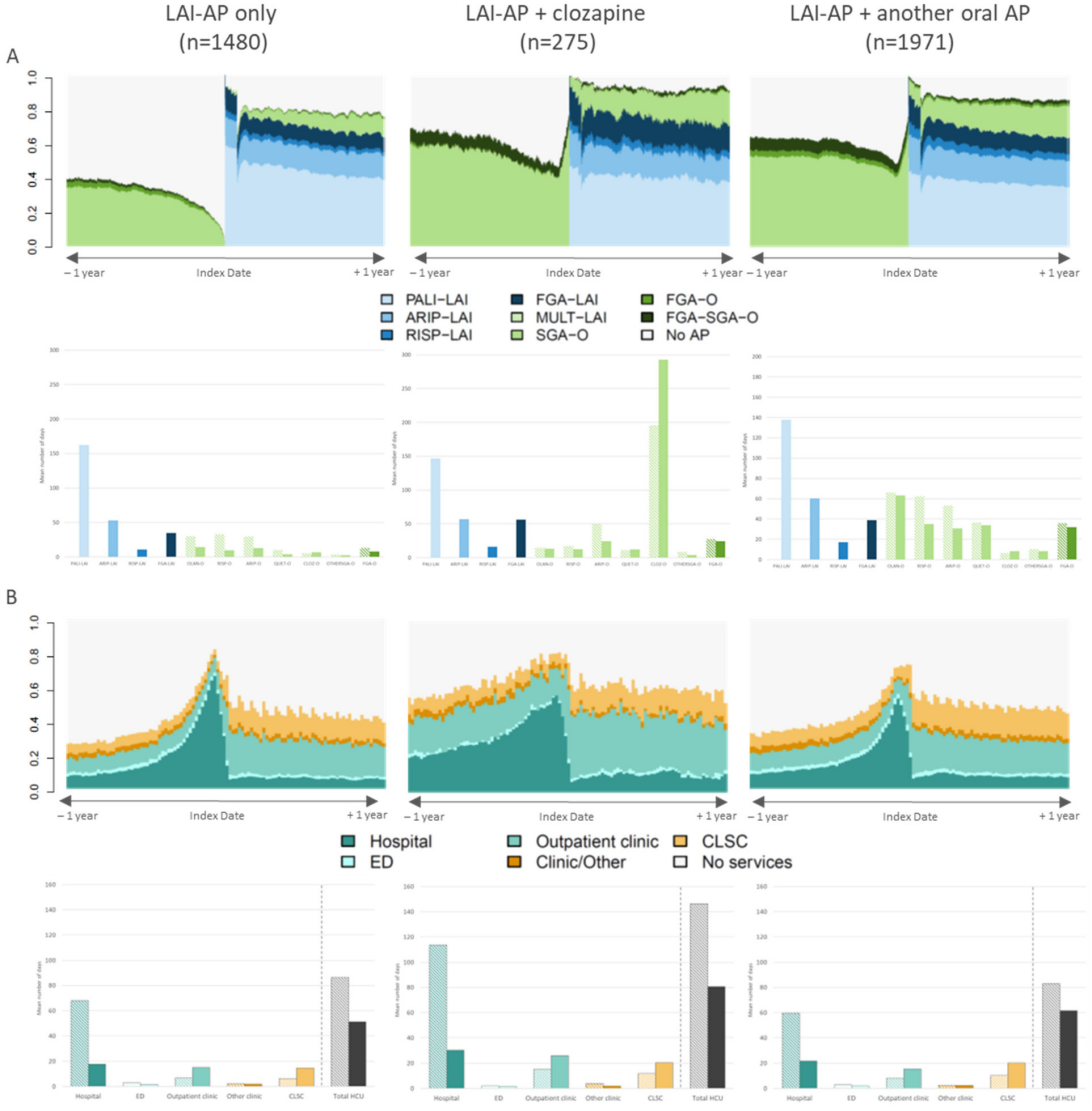
Antipsychotic trajectory (A) and healthcare use trajectory (B) before and after LAI-AP initiation among SCZ users by type of AP initiation (*n* = 3,726): state distribution plots^‡^ and bar charts.^§^

^‡^A state distribution plot shows the proportion of each state at each time unit (days for AP use trajectories, weeks for HCU trajectories) using a stacked bar chart, where the height of each segment represents the relative frequency of a specific state.

^§^Bar charts represent the overall number of days spent in each state before (hatched bar) and after (solid bar) index date.

Moreover, the AP use and healthcare use trajectories (as patterns of AP and healthcare use over time) before and after LAI-AP initiation stratified by groups (SCZ and non-SCZ) were presented using the visual representations offered by the State Sequence Analysis method, namely the state distribution plots (see Figure 1 for an example). These plots present, for each time unit, the proportion of patients in the group in each state (AP category or healthcare setting). State distribution plots summarize the proportion of patients in each treatment or healthcare-utilization state at every time point before and after LAI initiation. Because inpatient medication data were unavailable in the administrative pharmacy database, apparent reductions in antipsychotic exposure immediately prior to the index date likely correspond to hospitalization periods rather than true treatment discontinuation. To facilitate interpretation of the sequence analyses and to complement the priority-based visualization approach, additional bar charts present the mean number of days spent in each state before and after LAI initiation (Figures 1 and 2).

*The state distribution plot shows the proportion of each AP category selected using a stacked bar chart for each day, the height of each segment represents the relative frequency of a specific state.

^†^Bar charts represent the overall mean number of days spent in each state before (hatched bar) and after (solid bar) index date.

LAI-AP = long-acting injectable antipsychotics; SCZ = schizophrenia.

LAI-AP = long-acting injectable antipsychotics; SCZ = schizophrenia.

This method of analysis and visualization of trajectories have been used extensively by our team.^15,17–21^ Because of the limitation associated with the priority order, bar charts representing the mean number of days in each state are also presented as a complementary view of these AP and healthcare trajectories. The analyses were carried out using SAS 9.4 and the TraMineR package in R for the visualization of the trajectories.

## Results

The study cohort included 6221 patients (Supplemental Figure S1), 63.4% of whom were men. Patients in the SCZ group were more likely to be male (67% vs. 58%) and had a higher prevalence of low socioeconomic status compared to the non-SCZ group (85% vs. 73%). The average age at index date was 41.6 years, 26% were under 30 years old, and more than half were 39 years or younger (Table 1). The mental comorbidity profiles were significantly more common in the non-SCZ group. At LAI-AP treatment initiation, 55.7% of patients received paliperidone LAI, 21.5% received aripiprazole LAI, 6.9% received risperidone LAI, 15.6% received FGA LAI, and very few (0.2%) received more than one LAI-AP category. At index date, 40% received LAI-AP alone, 5% received LAI-AP with clozapine (more in the SCZ group), and the majority (55%) received LAI-AP with an oral AP (other than clozapine), among which 25% were the same molecule as the LAI-AP. Although paliperidone LAI was the most frequently prescribed LAI-AP at index date in both groups, its use was slightly higher among SCZ patients (56.4% vs. 54.6%), while non-SCZ patients had marginally higher use of aripiprazole and risperidone LAIs. Psychiatrists were the first prescribers of LAI-AP in most cases (more than 82%) (Table 1). Patients were also using other psychotropic medications in the year before index date, more frequently in the non-SCZ group: benzodiazepines (SCZ: 41.2%; non-SCZ: 43.3%), antidepressants (SCZ: 31.7%; non-SCZ: 36.8%), divalproex (SCZ: 15.2%; non-SCZ: 19.0%), lithium (SCZ: 7.1%; non-SCZ: 12.7%), and lamotrigine (SCZ: 2.4%; non-SCZ: 3.8%). The use of these medications remained relatively constant over time (Table 2(A)).

**Table 1.** Characteristics of the Study Population Among SCZ and Non-SCZ LAI-AP Users.

	All Users of LAI-AP (*n* = 6,221)	SCZ LAI-AP Users *n* = 3,726 (59.9%)	Non-SCZ LAI-AP Users *n* = 2,495 (40.1%)	*P*-Value^a^
Demographic and clinical characteristics at index date or during the 1-year baseline period				
Sex, *n* (%)				<.0001
Female	2,275 (36.6)	1,233 (33.1)	1,042 (41.8)	
Male	3,946 (63.4)	2,493 (66.9)	1,453 (58.2)	
Age, mean (SD)	41.6 (15.8)	41.2 (14.3)	42.2 (17.7)	.0133
Age, median (IQR)	39 (29-52)	39 (30-52)	39 (28-54)	.6865
Age group, n (%)				<.0001
< 30	1,645 (26.4)	907 (24.3)	738 (29.6)	
30-39	1,583 (25.4)	1,035 (27.8)	548 (22.0)	
40-49	1,105 (17.8)	692 (18.6)	413 (16.6)	
50-59	1,015 (16.3)	646 (17.3)	369 (14.8)	
≥ 60	873 (14.0)	446 (12.0)	427 (17.1)	
Low socioeconomic status, *n* (%)	4,986 (80.2)	3,167 (85.0)	1,819 (72.9)	<.0001
Comorbidity index (≥ 1), *n* (%)	1,768 (28.4)	975 (26.2)	793 (31.8)	<.0001
Hospitalization 1 month < index date, *n* (%)	4,858 (78.1)	2,912 (78.2)	1,946 (78.0)	.8830
Hospitalization 1 year < index date, *n* (%)	5,857 (94.2)	3,504 (94.0)	2,353 (94.3)	0.6604
Last psychotic diagnosis, *n* (%)				-
Schizophrenia (295, F20, F21, F23.2, F25)	3,726	3,726	-	
Non-affective	1,398	1,398		
Schizoaffective (295.7, F25)	643	643		
Unspecified/other type (295.8,.9, F20.8, .9)	1,685	1,685		
Bipolar disorder (296, F30, F31)	1,053 (39.7)	-	1,053 (39.7)	
Other psychosis (297, 298, F22-F24, F29)	1,390 (51.9)	-	1,390 (51.9)	
Missing	52 (2.1)	-	52 (2.1)	
Mental disorders, *n* (%)				
Depressive disorder	1,451 (23.3)	750 (20.1)	701 (28.1)	<.0001
Anxiety disorder	2,323 (37.3)	1,268 (34.0)	1,055 (42.3)	<.0001
Substance-use disorder	3,021 (48.6)	1,718 (46.1)	1,303 (52.2)	<.0001
Personality disorder	1,649 (26.5)	874 (23.5)	775 (31.1)	<.0001
Treatment characteristics at index date				
Index LAI-AP, *n* (%)				0.0162
Paliperidone LAI	3,465 (55.7)	2,130 (56.4)	1,362 (54.6)	
Aripiprazole LAI	1,340 (21.5)	773 (20.8)	567 (22.7)	
Risperidone LAI	431 (6.9)	234 (6.3)	197 (7.9)	
FGA LAI	971 (15.6)	607 (16.3)	364 (14.6)	
2 or more LAI	14 (0.2)	9 (0.2)	5 (0.2)	
Index AP, *n* (%)				<.0001
LAI-AP only	2,473 (39.8)	1,480 (39.7)	993 (39.8)	
LAI-AP + clozapine	335 (5.4)	275 (7.4)	60 (2.4)	
LAI-AP + oral AP other than Clozapine	3,413 (54.9)	1,971 (52.9)	1442 (57.8)	
LAI and oral: same molecule	866 (25.4)	485 (24.6)	381 (26.4)	
Specialty of the first prescriber, *n* (%)				
Psychiatrist	5,114 (82.2)	3,133 (84.1)	1,981 (79.4)	<.0001
General practitioner	1,053 (16.9)	566 (15.2)	487 (19.5)	
Other	54 (0.9)	27 (0.7)	27 (1.1)	

a Chi-2 test for categorical variables; t–test and Kruskal–Wallis for continuous variables.

**Table 2.** Medication and Healthcare Use Before and After LAI-AP Initiation Among SCZ and Non-SCZ LAI-AP Users.

	SCZ LAI-AP Users *n* = 3,679 (59.9%)			Non-SCZ LAI-AP Users *n* = 2,495 (40.1%)			SCZ vs. Non-SCZ	
	Before Index Date	After Index Date	*P*-Value^a^	Before Index Date	After Index Date	*P*-Value^a^	*P*-Value^b^	*P*-Value^c^
A. Drug treatment (at least one claim at the community pharmacy)								
LAI-AP, *n* (%)								
Paliperidone LAI		2,273 (61.0)			1461 (58.6)			.0535
Aripiprazole LAI	-	941 (25.2)	-	-	702 (28.1)	-	-	.0115
Risperidone LAI		265 (7.1)			216 (8.7)			.0253
FGA LAI		727 (19.5)			405 (16.2)			.0010
Oral AP at least one claim, *n* (%)								
Risperidone	1,103 (29.6)	733 (19.7)	<.0001	759 (30.4)	578 (23.2)	<.0001	.4899	.0009
Olanzapine	1,069 (28.7)	829 (22.2)	<.0001	644 (25.8)	482 (19.3)	<.0001	.0127	.0055
Aripiprazole	1,034 (27.8)	745 (20.0)	<.0001	685 (27.4)	553 (22.2)	<.0001	.7980	.0390
Quetiapine	485 (13.0)	384 (10.3)	<.0001	363 (14.6)	288 (11.5)	<.0001	.0843	.1234
Clozapine	364 (9.8)	436 (11.7)	<.0001	76 (3.0)	130 (5.2)	<.0001	<.0001	<.0001
Ziprasidone	92 (2.5)	43 (1.2)	<.0001	50 (2.0)	21 (0.8)	<.0001	.2286	.2314
Paliperidone	12 (0.3)	8 (0.2)	0.3173	7 (0.3)	7 (0.3)	1.0000	.7713	.6037
FGA	594 (15.9)	566 (15.2)	0.2132	281 (11.3)	315 (12.6)	.0481	<.0001	.0045
Other medication, *n* (%)								
Lithium use	266 (7.1)	251 (6.7)	0.1470	317 (12.7)	278 (11.1)	.0005	<.0001	<.0001
Antidepressant use	1,180 (31.7)	1,194 (32.0)	0.5770	918 (36.8)	961 (38.5)	.0518	<.0001	<.0001
Divalproex use	568 (15.2)	559 (15.0)	0.4862	475 (19.0)	457 (18.3)	.1699	<.0001	.0005
Lamotrigine use	91 (2.4)	87 (2.3)	0.6171	95 (3.8)	90 (3.6)	.5078	.0019	.0031
Benzodiazepine use	1,537 (41.2)	1506 (40.4)	0.2045	1081 (43.3)	1037 (41.6)	.0389	.1041	.3682
B. Mean number of drugs claimed at the pharmacy (*SD*)								
AP prescription drugs	29.7 (46.3)	39.7 (42.2)	<.0001	23.3 (40.0)	32.7 (35.6)	<.0001	.0001	<.0001
All prescription drugs	110.1 (213.7)	153.5 (237.3)	<.0001	116.3 (230.1)	156.2 (236.7)	<.0001	.2787	.6607
C. Mean number of healthcare use (SD)								
Hospitalization days	67.0 (83.6)	20.8 (47.9)	<.0001	51.5 (64.6)	16.3 (39.4)	<.0001	<.0001	<.0001
ED visits	2.8 (4.2)	1.7 (3.4)	<.0001	3.5 (5.6)	1.9 (4.5)	<.0001	<.0001	.0617
Outpatient visits	8.0 (13.2)	16.1 (21.9)	<.0001	7.2 (14.5)	13.5 (16.7)	<.0001	.0335	<.0001
Primary care clinic	2.5 (8.0)	2.2 (6.2)	0.1235	2.6 (4.9)	3.0 (6.5)	.0012	.4295	<.0001
CLSC	8.9 (24.0)	18.1 (34.2)	<.0001	8.8 (16.9)	16.9 (33.1)	<.0001	.9331	.1569
All HCU	89.2 (83.8)	58.9 (62.3)	<.0001	75.6 (67.5)	51.5 (55.0)	<.0001	<.0001	<.0001

a McNemar test for paired categorical variables; Paired t–test for continuous variables.

b Before index date. Chi–2 test for categorical variables; t–test and Kruskal–Wallis for continuous variables.

c After index date. Chi–2 test for categorical variables; t–test and Kruskal–Wallis for continuous variables.

### Antipsychotic Utilization Trajectories

#### SCZ versus non-SCZ

The antipsychotic use trajectories before and after index date are summarized in Table 2(A)-(B) and represented graphically in Figure 1(A) using state distribution plots and bar charts. The SCZ group had significantly higher clozapine use before (9.8% vs. 3.0%) and after (11.7% vs. 5.2%) index date than the non-SCZ group (Table 2(A)), and higher use of oral FGA before (15.9% vs. 11.3%) and after (15.2% vs. 12.6%) LAI-AP initiation. Both groups show a significant decrease in oral AP use, except for clozapine, which increased, and oral FGA, which remained constant (Table 2(A)-(B), Figure 1(A)).

#### LAI only versus LAI + clozapine versus LAI + another oral AP

Within the SCZ group, at index date, 40% received an LAI only, 7% received an LAI and were exposed to clozapine, but a majority 53% received an LAI and were exposed to another oral AP. The clozapine subgroup was younger, had more males, had low socioeconomic status in a higher proportion, but had less co-occurring mental disorders than the other 2 subgroups (Supplemental Table S1). This subgroup received a higher proportion of FGA-LAI at the index date and a higher proportion of psychiatrists as the first prescriber. The clozapine subgroup used more co-occurring medications than the other groups, except for benzodiazepines, both before and after the index date (Table 3). Figure 2(A) represents graphically the AP treatment trajectories before and after index for all 3 subgroups. These observations remained essentially the same within the non-SCZ group (Supplemental Tables 2-3).

**Table 3.** Medication and Healthcare Use Before and After LAI-AP Initiation Among SCZ Users by Type of AP Initiation (*n* = 3,726).

	LAI Only (*n* = 1,480)			LAI + Clozapine (*n* = 275)			LAI + Another Oral AP (*n* = 1,971)				
	Before Index Date	After Index Date	*P*-Value^a^	Before Index Date	After Index Date	*P*-Value	Before Index Date	After Index Date	*P*-Value	*P*-Value^b^	*P*-Value^c^
A. Drug treatment (at least one claim at the community pharmacy)											
LAI-AP, *n* (%)											
Paliperidone LAI		1,000 (67.6)			148 (53.8)			1125 (57.1)			< .0001
Aripiprazole LAI	-	360 (24.3)	-	-	65 (23.6)	-	-	516 (26.2)		-	.3766
Risperidone LAI		79 (5.3)			16 (5.8)			170 (8.6)			.0007
FGA LAI		259 (17.5)			63 (22.9)			405 (20.6)			.0276
Oral AP at least one claim, *n* (%)											
Risperidone	361 (24.4)	171 (11.6)	< .0001	35 (12.7)	27 (9.8)	.1944	707 (35.9)	535 (27.1)	< .0001	< .0001	< .0001
Olanzapine	323 (21.8)	169 (11.4)	< .0001	37 (13.4)	33 (12.0)	.5164	709 (36.0)	627 (31.8)	<. 0001	< .0001	< .0001
Aripiprazole	313 (21.2)	190 (12.8)	< .0001	78 (28.4)	58 (21.1)	.0039	643 (32.6)	497 (25.2)	< .0001	< .0001	< .0001
Quetiapine	97 (6.6)	50 (3.4)	< .0001	22 (8.0)	17 (6.2)	.2253	366 (18.6)	317 (16.1)	.0008	< .0001	< .0001
Clozapine	47 (2.8)	60 (4.0)	.0415	249 (90.6)	265 (96.4)	.0077	73 (3.7)	111 (5.6)	.0025	< .0001	< .0001
Ziprasidone	27 (1.8)	8 (0.5)	<. 0001	-	-	-	62 (3.2)	33 (1.7)	< .0001	.0145	.0067
Paliperidone	-	-	-	-	-	-	-	-	-	-	-
FGA	130 (8.8)	105 (7.1)	.0488	58 (21.1)	63 (22.9)	.4658	406 (20.6)	398 (20.2)	.6431	< .0001	< .0001
Other medication, *n* (%)											
Lithium use	84 (5.7)	79 (5.3)	.4349	43 (15.6)	37 (13.4)	.0578	139 (7.0)	135 (6.8)	.5930	<. 0001	< .0001
Antidepressant use	360 (24.3)	391 (26.4)	.0541	116 (42.2)	106 (38.6)	.0956	704 (35.7)	697 (35.4)	.7021	< .0001	< .0001
Divalproex use	162 (11.0)	157 (10.6)	.5078	68 (24.7)	68 (24.7)	1.0000	338 (17.2)	334 (17.0)	.6799	< .0001	< .0001
Lamotrigine use	27 (1.8)	22 (1.5)	.3359	22 (8.0)	22 (8.0)	1.0000	42 (2.1)	43 (2.2)	.8474	< .0001	< .0001
Benzodiazepine use	455 (30.7)	475 (32.1)	.2041	109 (39.6)	117 (42.6)	.2059	973 (49.4)	914 (46.4)	.0008	< .0001	< .0001
B. Mean number of drugs claimed at the pharmacy (SD)											
AP prescription drugs	14.6 (28.3)	21.1 (23.4)	< .0001	52.9 (70.1)	77.8 (52.1)	< .0001	37.7 (49.5)	48.4 (46.4)	< .0001	< .0001	< .0001
All prescription drugs	61.4 (180.3)	92.2 (181.7)	< .0001	192.1 (148.9)	278.8 (235.6)	< .0001	135.2 (200.4)	182.1 (239.7)	< .0001	< .0001	< .0001
C. Mean number of healthcare use (SD)											
Hospitalization days	68.1 (64.4)	17.8 (15.6)	< .0001	113.8 (99.3)	30.4 (61.9)	< .0001	59.7 (82.2)	21.8 (49.3)	< .0001	< .0001	< .0001
ED visits	2.9 (3.8)	1.6 (3.3)	< .0001	1.9 (1.6)	1.8 (3.8)	.5402	2.8 (4.6)	1.9 (3.4)	< .0001	< .0001	.0011
Outpatient visits	6.7 (11.5)	15.1 (22.3)	< .0001	15.0 (20.2)	26.0 (28.7)	< .0001	8.0 (12.8)	15.4 (20.1)	< .0001	< .0001	< .0001
Primary care clinic	2.2 (6.5)	2.0 (5.5)	.4582	3.8 (15.4)	2.0 (4.7)	.0471	2.5 (7.4)	2.4 (6.9)	.7190	.0009	.0120
CLSC	6.4 (17.6)	14.8 (27.3)	< .0001	11.9 (28.6)	20.5 (40.8)	< .0001	10.3 (27.2)	20.2 (37.5)	< .0001	0.0489	<.0001
All HCU	86.4 (73.3)	51.3 (55.6)	< .0001	146.4 (112.0)	80.7 (72.1)	< .0001	83.3 (83.7)	61.6 (64.6)	< .0001	< .0001	< .0001

a McNemar test for paired categorical variables; Paired t-test for continuous variables.

b Before index date. Chi-2 test for categorical variables; Kruskal-Wallis for continuous variables.

c After index date. Chi-2 test for categorical variables; Kruskal-Wallis for continuous variables.

### Healthcare Utilization Trajectories

#### SCZ versus non-SCZ

In the year preceding the LAI initiation, SCZ patients had a higher average number of hospitalization days (67.0 vs. 51.5), reflecting greater illness severity or instability (Table 2(C), Figure 3). Both groups exhibited substantial reductions in hospitalization days in the year post-LAI-AP, with SCZ patients showing a 69% decrease (from 67.0 to 20.8 days) and non-SCZ, a 68% decrease (from 51.5 to 16.3 days). Both groups increased substantially their outpatient visits as well as CLSC's support after index date, although primary care visits remained stable and low (Table 2(C), Figure 1(B)).

#### LAI only versus LAI + clozapine versus LAI + another oral AP

The clozapine subgroup among patients with SCZ represents the most severe group, as shown by the large number of days spent in hospital before the index date, which is almost twice that of the other subgroups (Table 3(C), Figure 2(B)). This subgroup also used a large amount of healthcare services and claimed a larger number of drugs at the pharmacy (Table 3(B)-(C)). As before, the mean number of hospital days decreased dramatically in the year after LAI initiation, and this was true for all subgroups (from 63% to 73% decrease). The 3 subgroups also increased substantially their outpatient visits and CLSC's visits after index date (Table 4(C), Figure 2(B)). Once again, these observations remained essentially unchanged within the non-SCZ group (Supplemental Table S3, Supplemental Figure S2).

## Discussion

This study of 6221 patients initiating LAI-APs in Québec reveals notable differences between individuals with a final diagnosis of SCZ and those classified under other psychotic disorders (non-SCZ). While both groups benefited from reduced healthcare utilization after LAI-AP initiation, their baseline characteristics, clinical profiles, and trajectories of service use suggest distinct clinical needs and treatment contexts.

Patients in the SCZ group were more likely to be male and had a higher prevalence of low socioeconomic status compared to the non-SCZ group. These differences may reflect the chronic and disabling nature of SCZ, often associated with earlier onset and greater functional impairment. Comorbidity profiles also diverged: depressive, anxiety, substance use disorders, and personality disorders were significantly more common in the non-SCZ group, suggesting a greater psychopathological complexity.

Although paliperidone LAI was the most frequently prescribed LAI-AP in both groups, its use was only slightly higher among SCZ patients (61.0% vs. 58.6%), while non-SCZ patients had marginally higher use of aripiprazole and risperidone LAIs. The SCZ group had higher clozapine and FGA oral AP use, which may indicate more treatment-resistant or more severe cases. Differences in prescribing patterns may reflect diagnostic preferences, tolerability concerns, or clinical expectations of adherence.

The use of mood stabilizers (lithium, divalproex, lamotrigine) and benzodiazepines was common in both groups, though slightly more frequent in the non-SCZ population, consistent with their broader affective and anxiety-related comorbidities. Antidepressant use was similar between groups and remained stable before and after LAI initiation.

### Healthcare Utilization Trajectories

These findings suggest that while both SCZ and non-SCZ patients benefit from LAI-AP treatment in terms of reduced hospital use and improved care continuity, the clinical presentation and service needs of the 2 groups differ significantly. SCZ patients appear to represent a more socioeconomically vulnerable and clinically severe population, whereas non-SCZ patients may present with more diverse psychiatric comorbidities requiring complex pharmacological and non-pharmacological management, at least for substance use disorder comorbidities.

Recognizing these differences is essential for tailoring interventions and optimizing treatment strategies. The effectiveness of LAI-APs across these diagnostic categories supports their continued use, but nuanced clinical pathways may be needed to address the specific needs of each subgroup, including psychosocial interventions and support, adherence monitoring, and coordinated and integrated outpatient care.

Compared with our earlier Québec registry studies^10^ conducted on cohorts from 2008 to 2012, the present analysis includes a substantially larger and more recent population and confirms the robustness of the observed reductions in hospitalization and emergency service utilization following LAI initiation. In the previous cohort, hospitalization days decreased by approximately two-thirds following LAI initiation, whereas in the present study, the reduction reached nearly 3-quarters in certain subgroups, suggesting not only confirmation but a strengthening of the observed trend over time. In addition, the present study extends previous work by using trajectory-based analyses to characterize patterns of healthcare utilization and antipsychotic exposure across SCZ and non-SCZ psychosis subgroups. The observed increase in outpatient and community-based follow-up, including CLSC utilization, may reflect closer integration into ambulatory care programs and improved continuity of care, although this interpretation should remain cautious given the possible influence of community treatment orders in Québec.

In addition to our findings, recent large registry studies and meta-analyses consistently show that LAI-APs are associated with lower relapse rates than their oral counterparts, with paliperidone 3-month LAI, aripiprazole LAI, and olanzapine LAI repeatedly ranking among the most effective options for relapse prevention.^22–25^ Furthermore, network meta-analyses and randomized controlled trials indicate that both LAI and oral formulations of paliperidone, aripiprazole, and olanzapine are superior to placebo for preventing relapse, although direct head-to-head differences between oral and injectable forms remain generally modest. The slight but meaningful advantage observed for LAIs in real-world settings is largely attributable to improved adherence, a critical factor in populations where non-adherence drives relapse risk and healthcare utilization.^22–30^

Within this broader evidence base, our results align with and extend previous observations, showing that at a population level, LAI initiation is followed by a marked reduction in hospitalization and greater stabilization of outpatient care, which likely reflect the clinical benefits of more consistent antipsychotic exposure.^1–6,31,32^

As shown in our data, anecdotally 14 patients received 2 LAI-AP concurrently. While it is theoretically possible, this is not standard practice, and must be done with caution, with full awareness of the indication, and ideally in a second-line setting or highly resistant cases. Indeed, a 2024 systematic review^31^ found that combinations of 2 LAIs have been used: for example, Paliperidone palmitate + Aripiprazole monohydrate were reported in multiple cases. Another recent retrospective cohort study^32^ in treatment-resistant SCZ receiving Clozapine found that augmentation with an LAI was associated with a longer time to rehospitalisation than Clozapine alone or with oral antipsychotics only.

### Limitations

First, the lack of comparator group is a major limitation. Indeed, the important decrease in hospital days before and after LAI-AP initiation, as for most mirror-image studies, may be due at least partly to the “regression to the mean” effect.^33–35^ This statistical phenomenon can give the appearance of real change where there is actually just natural variation in repeated data. It happens when unusually large (or small) measurements (like an important hospital rate or length of stay) tend to be followed by measurements that are closer to the mean. The best way to control for this effect is the use of an appropriate comparator group. However, the selection of such a comparator group was difficult in the current study. Indeed, one might wonder whether an ideal control population from the registry could not have consisted of patients who also switched their oral antipsychotic medication to another oral medication at the Index date. However, the indication to switch from one oral formulation to a long-acting antipsychotic is truly a decision regarding medication adherence, while switching from one oral antipsychotic medication to another oral antipsychotic medication may more closely reflect a need to switch due to side effects or an insufficient or incomplete clinical response. Nevertheless, the decrease in hospital days before and after the index date was so pronounced (nearly 70% in both SCZ and non-SCZ groups) that we are confident that it is unlikely to be explained solely by the regression to the mean effect, even in the absence of a control group. The study is also limited by the period accessible to the registry, which ended in 2017. Other formulations were just emerging or were not yet marketed. The study's limitations are also related to the registry itself, such as the diagnoses recorded in patient records, which would merit more detailed examination, and by the absence of socioeconomical characteristics (such as race, income). As in many real-world observational studies, antipsychotic polypharmacy and concomitant psychotropic medication use complicate the interpretation of treatment trajectories. A substantial proportion of patients received LAIs in combination with oral antipsychotics, which may reflect cross-titration strategies, partial adherence, treatment resistance, symptom severity, or attempts to stabilize patients during periods of clinical transition. Administrative databases cannot fully capture the clinical rationale underlying these prescribing decisions. Consequently, the observed treatment patterns should be interpreted as reflecting routine clinical practice rather than standardized pharmacological strategies.

Administrative healthcare databases provide valuable population-level longitudinal information with minimal attrition and strong external validity. However, they are limited by the accuracy of diagnostic coding, the absence of detailed clinical assessments, incomplete inpatient medication information, and the limited availability of certain sociodemographic variables such as ethnicity, race, psychosocial functioning, and individual-level socioeconomic measures.

## Conclusion

This type of registry study provides a real-world portrait of prescribing practices across a large population of clinicians and patients within the Québec healthcare system. In contrast to highly controlled randomized trials, such observational data capture the complexity, heterogeneity, and ecological reality of routine psychiatric practice. One might conceptualize registry-based mirror studies as a form of “healthcare system selfie,” capturing a naturalistic and ecologically grounded portrait of prescribing behaviours at the population level. In patients with SCZ, schizoaffective disorder, or another psychotic disorder who at some point required a switch to a long-acting antipsychotic, the initiation of a long-acting injectable significantly reduced the use of healthcare resources. As we documented and modelled 10 years ago with a mirror study using an identical registry, this could translate into lower overall costs compared to previously administered oral antipsychotics in Québec. A pharmacoeconomic analysis using a comparator through target trial simulation design with propensity scores will be needed in the future to confirm this. The results of our study support the recommendations of the Canadian Psychiatric Association and the Québec Association of Psychiatrists regarding the use of LAIs particularly based on the QAAPAPLE Algorithm which is currently being updated.^9,36–40^

## Supplemental Material

sj-docx-1-cpa-10.1177_07067437261462697 - Supplemental material for Healthcare Pattern of Use Before and After Initiating a Long-Acting Antipsychotic Among a Cohort of 6221 Patients With a History of Psychosis Supplemental material, sj-docx-1-cpa-10.1177_07067437261462697 for Healthcare Pattern of Use Before and After Initiating a Long-Acting Antipsychotic Among a Cohort of 6221 Patients With a History of Psychosis by Emmanuel Stip, MD, MSc, FRCP, Josiane Courteau, PhD, Sébastien Brodeur, MD, MSc, FRCP, Yohann M. Chiu, PhD, Marie-Josée Fleury, PhD, Alain Lesage, MD, MPhil and Marc-André Roy, MD, FRCP in The Canadian Journal of Psychiatry
