# Supplementary material for: Healthcare Pattern of Use Before and After Initiating a Long-Acting Antipsychotic Among a Cohort of 6221 Patients With a History of Psychosis
Source: Can J Psychiatry. 2026 Jun 29:07067437261462697. Online ahead of print. doi: 10.1177/07067437261462697 (PMC13314648; doi:10.1177/07067437261462697)
Supplement: sj-docx-1-cpa-10.1177_07067437261462697 - Supplemental material for Healthcare Pattern of Use Before and After Initiating a Long-Acting Antipsychotic Among a Cohort of 6221 Patients With a History of Psychosis [file sj-docx-1-cpa-10.1177_07067437261462697.docx]

**Supplementary Figure 1.** Study cohort

**
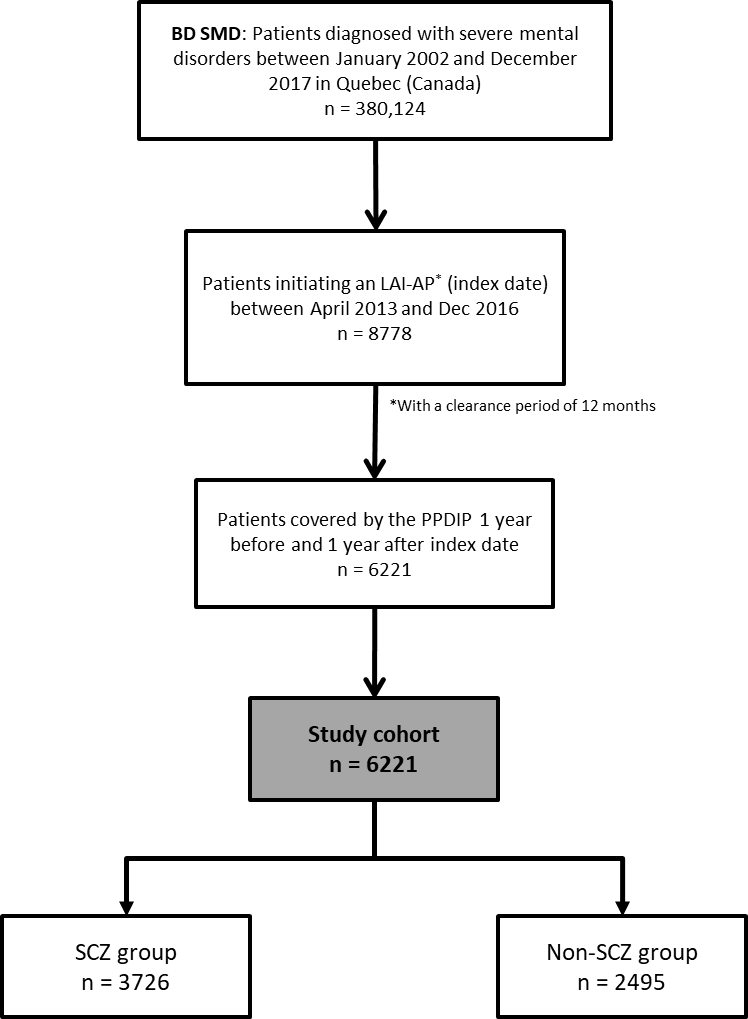
**

**Supplementary Figure 2**. Antipsychotic trajectory (A) and healthcare use trajectory (B) before and after LAI-AP initiation among non-SCZ users by type of AP initiation (n=2495): state distribution plots^[[1]](#footnote-1)^ and bar charts^[[2]](#footnote-2)^


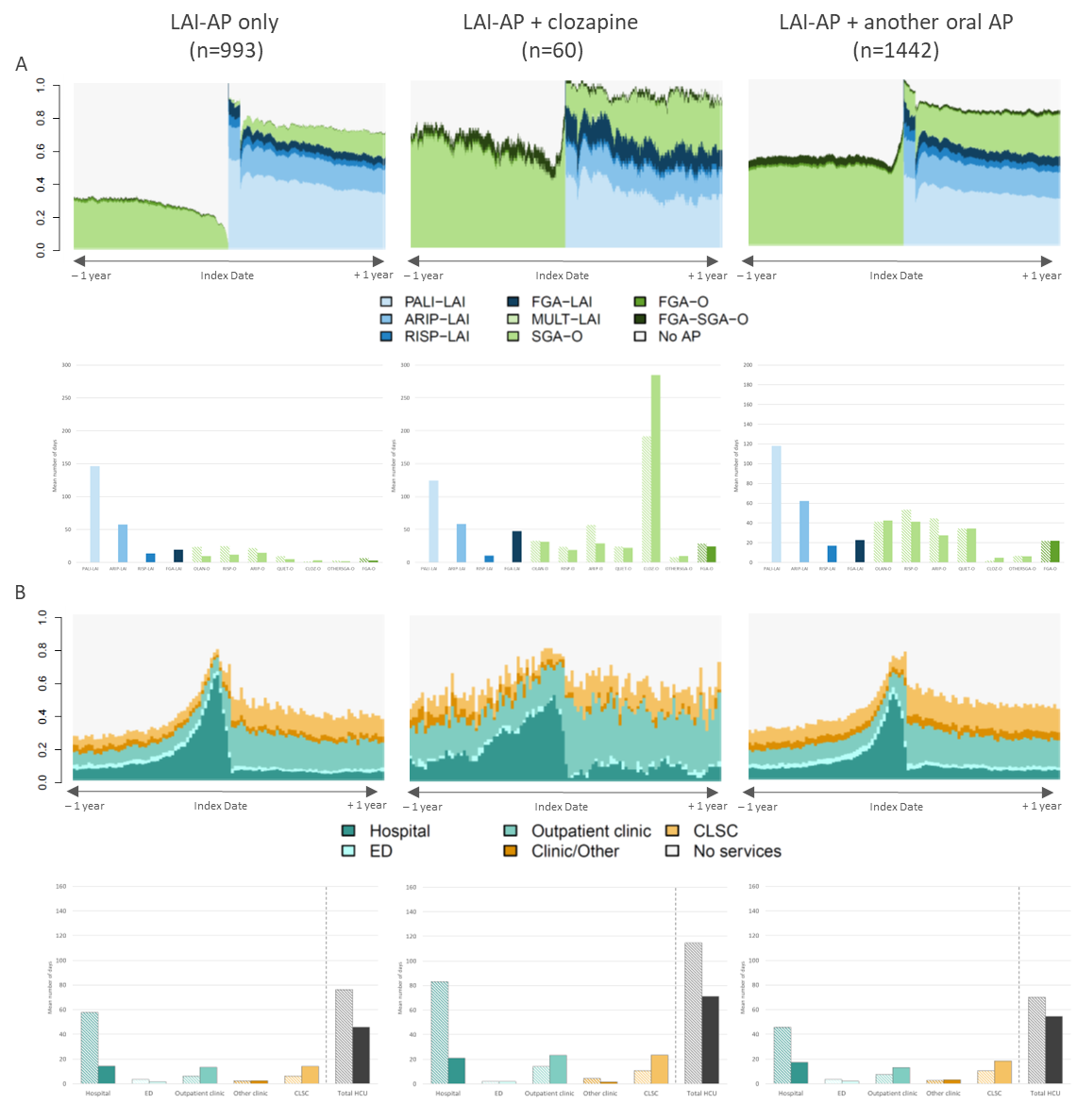


**Supplementary Table 1**. Characteristics of the SCZ LAI-AP users according to type of AP initiation (n=3726)

|  | LAI only  n=1480, 39.7% | LAI + Clozapine  n=275, 7.4% | LAI + another oral AP  n=1971, 52.9% | p-value^[[3]](#footnote-3)^ |
| --- | --- | --- | --- | --- |
| Demographic and clinical characteristics at index date or during the 1-year baseline period | | | | |
| Sex, n (%)  Female  Male | 519 (35.1)  961 (64.9) | 69 (25.1)  206 (74.9) | 645 (32.7)  1326 (67.3) | 0.0048 |
| Age, mean (SD) | 40.9 (14.6) | 38.8 (12.2) | 41.5 (14.1) | 0.0058 |
| Age, median (IQR) | 38 (29 – 51) | 36 (29 – 48) | 39 (30 – 52) | 0.0105 |
| Low socioeconomic status, n (%) | 1195 (80.7) | 248 (90.2) | 1724 (87.5) | <.0001 |
| Comorbidity index (≥ 1), n (%) | 359 (24.3) | 62 (22.6) | 554 (28.1) | 0.0142 |
| Hospitalization 1 month < index date, n (%) | 1184 (80.0) | 200 (72.7) | 1528 (77.5) | 0.0169 |
| Hospitalization 1 year < index date, n (%) | 1423 (96.2) | 249 (90.6) | 1832 (93.0) | <.0001 |
| Mental disorders, n (%)  Depressive disorder  Anxiety disorder  Substance-use disorder  Personality disorder | 314 (21.2)  511 (34.5)  716 (48.4)  359 (24.3) | 42 (15.3)  89 (32.4)  101 (36.7)  49 (17.8) | 394 (20.0)  668 (33.9)  901 (45.7)  466 (23.6) | 0.0763  0.7711  0.0016  0.0660 |
| Treatment characteristics at index date | | | | |
| Index LAI-AP, n (%)  Paliperidone LAI  Aripiprazole LAI  Risperidone LAI  FGA LAI or 2 or more LAI | 942 (63.6)  265 (17.9)  63 (4.3)  210 (14.2) | 142 (51.6)  61 (22.2)  14 (5.1)  58 (21.1) | 1019 (51.7)  447 (22.7)  157 (8.0)  348 (17.7) | < .0001 |
| Specialty of the first prescriber, n (%)  Psychiatrist  General practitioner/other MD | 1227 (82.9)  253 (17.1) | 257 (93.4)  18 (6.6) | 1649 (83.7)  322 (16.3) | <.0001 |

**Supplementary Table 2**. Characteristics of the non-SCZ LAI-AP users according to type of AP initiation (n=2495)

|  | LAI only  n=993, 39.8% | LAI + Clozapine  n=60, 2.4% | LAI + another oral AP  n=1442, 57.8% | p-value^[[4]](#footnote-4)^ |
| --- | --- | --- | --- | --- |
| Demographic and clinical characteristics at index date or during the 1-year baseline period | | | | |
| Sex, n (%)  Female  Male | 427 (43.0)  566 (57.0) | 20 (33.3)  40 (66.7) | 595 (41.3)  847 (58.7) | 0.2826 |
| Age, mean (SD) | 41.6 (16.9) | 38.0 (13.5) | 42.8 (18.4) | 0.0466 |
| Age, median (IQR) | 39 (28 – 54) | 35 (27 – 49) | 39 (28 – 54) | 0.2204 |
| Comorbidity index (≥ 1), n (%) | 298 (30.0) | 13 (21.7) | 482 (33.4) | 0.0482 |
| Hospitalization 1 month < index date, n (%) | 789 (79.5) | 42 (70.0) | 1115 (77.3) | 0.1459 |
| Mental disorders, n (%)  Depressive disorder  Anxiety disorder  Substance-use disorder  Personality disorder | 264 (26.6)  410 (41.3)  541 (54.5)  292 (29.4) | 13 (21.7)  20 (33.3)  23 (38.3)  15 (25.0) | 424 (29.4)  625 (43.3)  739 (51.2)  468 (32.4) | 0.1679  0.2193  0.0271  0.1646 |
| Treatment characteristics at index date | | | | |
| Index LAI-AP, n (%)  Paliperidone LAI  Aripiprazole LAI  Other LAI | 611 (61.5)  208 (21.0)  174 (17.5) | 33 (55.0)  12 (20.0)  15 (25.0) | 718 (49.8)  347 (24.1)  377 (26.1) | < .0001 |
| Specialty of the first prescriber, n (%)  Psychiatrist  General practitioner/other MD | 801 (80.7)  192 (19.3) | 54 (90.0)  6 (10.0) | 1126 (78.1)  316 (21.9) | 0.0366 |

Chi-2 test for categorical variables; t-test and Kruskal-Wallis for continuous variables.

1. A state distribution plot shows the proportion of each state at each time unit (days for AP use trajectories, weeks for HCU trajectories) using a stacked bar chart, where the height of each segment represents the relative frequency of a specific state. [↑](#footnote-ref-1)
2. Bar charts represent the overall number of days spent in each state before (hatched bar) and after (solid bar) index date. [↑](#footnote-ref-2)
3. Chi-2 test for categorical variables; t-test and Kruskal-Wallis for continuous variables. [↑](#footnote-ref-3)
4. [↑](#footnote-ref-4)
